# Understanding and Managing Pediatric Urinary Tract Infections in Vesicoureteral Reflux: Insights Into Pathophysiology and Care

**DOI:** 10.7759/cureus.76144

**Published:** 2024-12-21

**Authors:** Alaa S Alyasi, Deema Badr Alsaad, Eman Mohamed Alshammary, Aljallal Ali Abdulrahman, Mashniyyah Hassan Ghazwani, Mohammed Jafar Almuayrifi, Shrooq Saad Alharbi, Eiman Mohammed Ali Alali, Mohamad Aiman Daghestani, Shahad Mohammed Alrefaei, Hamad Khalid H Alolaywi

**Affiliations:** 1 Pediatrics and Neonatology, Maternal and Child Health Care Center, Tabuk, SAU; 2 General Pediatrics, King Abdullah Bin Abdulaziz University Hospital, Riyadh, SAU; 3 General Practice, King Salman Specialized Hospital, Ha'il, SAU; 4 General Practice, General Directorate for Prisons - Malaz Prison, Riyadh, SAU; 5 Pediatrics, Maternity and Children Hospital, Al-Jouf, SAU; 6 General Practice, King Salman Bin Abdulaziz Medical City, Madinah, SAU; 7 Pediatrics, Taibah University, Madinah, SAU; 8 Respiratory Medicine, Weber State University, Al-Ahsa, SAU; 9 General Practice, Sulaiman Al Rajhi University, Bukayriyah, SAU; 10 Pediatrics, King Saud Bin Abdulaziz University for Health Sciences, Riyadh, SAU; 11 General Practice, King Saud Bin Abdulaziz University for Health Sciences, Riyadh, SAU

**Keywords:** continuous antibiotic prophylaxis, cost-effectiveness, pediatric, urinary tract infection, vesicoureteral reflux

## Abstract

Vesicoureteral reflux (VUR) is a pediatric condition identified by the backward flow of urine from the bladder to one or both ureters and kidneys, predisposing patients to recurrent urinary tract infections (UTIs) and kidney scarring. Continuous antibiotic prophylaxis has long been a mainstay of management aimed at preventing recurrent UTIs and resulting renal damage. This review critically discusses the evidence supporting the utilization of antibiotic prophylaxis in VUR, with a focus on its efficacy, safety, long-term outcomes, and future directions in management. The literature reveals that continuous antibiotic use as a prophylactic measure minimizes the possibility of having recurrent UTIs in VUR children, especially in high-grade reflux children. However, the overall benefit of continuous antibiotic prophylaxis in protecting against kidney scarring remains controversial. Furthermore, concerns about antibiotic resistance, adverse drug reactions, and the psychosocial burden on families have led to a reevaluation of this option’s role in managing VUR. Emerging evidence supports the role of non-antibiotic interventions and the potential of surgical management in select cases. Future research should focus on identifying criteria of patients who would benefit most from continuous antibiotic prophylaxis and on developing novel therapeutic approaches to minimize the need for prolonged antibiotic use.

## Introduction and background

Vesicoureteral reflux (VUR) happens when urine passes from the bladder to the ureter and then to the kidney due to a malfunctioning vesicoureteric junction. This junction is critical in maintaining a one-way urine flow from the ureters to the bladder. It consists of a combination of structural and functional components designed to prevent the retrograde flow of urine (reflux) [1]. VUR occurs in 1.3% of healthy children, but 36-56% of children having urinary tract infections (UTIs) have it, especially when UTIs happen at a younger age. Improvements in prenatal imaging techniques have enabled the detection of asymptomatic patients with VUR during postnatal evaluations for hydronephrosis [2,3]. Additionally, the likelihood of renal damage increases with the frequency of UTIs. Kidney scarring was observed in 26% of children who experienced no recurrence, 38% and 80% of whom experienced a single recurrence and more than one recurrence of UTIs, respectively [4-6].

Continuous antibiotic prophylaxis (CAP) is commonly regarded as an innovative conservative treatment for VUR. It effectively protects against recurrent UTIs and the subsequent development of new kidney scarring. The Randomized Intervention for Children with Vesicoureteral Reflux (RIVUR) trial demonstrated that giving trimethoprim and sulfamethoxazole (TMP-SMX) continuously reduced UTI occurrence by nearly half in patients aged 2-71 months and diagnosed with VUR following their initial UTI. However, it did not completely prevent the occurrence of new kidney scarring [7].

In the last decade, mounting evidence has opposed the previously accepted approaches for preventing VUR-associated UTIs. There are conflicting recommendations associated with these traditional approaches, including prophylactic antibiotics. This review aims to comprehensively evaluate the current efficacy, safety, and limitations of using continuous antibiotics as a protective measure against UTIs in children with VUR. Additionally, it explores potential future considerations related to this treatment approach.

## Review

Methods

In August 2024, a comprehensive search was conducted across PubMed, Scopus, and Web of Science using keywords such as “antibiotic prophylaxis”, “vesicoureteral reflux (VUR)”, “urinary tract infection (UTI)”, “pediatric OR children”, "scarring DMSA", and Medical Subject Headings (MeSH). Additionally, key references were identified from the bibliographies of relevant studies. To narrow down the focus to specific study types, filters were applied for clinical trials, meta-analyses, randomized control trials, and systematic reviews, covering a period from August 2014 to August 2024. We followed a structured approach to ensure a comprehensive synthesis of the literature. The methods involved identifying relevant publications by using specific keywords in major databases, critically appraising the findings from diverse studies, and integrating insights to provide a coherent overview. The conclusions were drawn based on the collective interpretation of evidence from these studies, emphasizing key trends, gaps, and implications in the context of the topic.

Pathophysiology of VUR

The reflux occurs due to the immaturity or failure of the mechanisms that normally prevent reflux, which can be caused by anatomical or functional abnormalities [1]. Primary VUR is a prevalent condition often linked to congenital anomalies in the vesicoureteral junction (VUJ) due to abnormal embryological development. The VUJ serves as the division between the low-pressure upper tract and the high-pressure lower tract.

The most widely accepted explanation for an effective antireflux mechanism involves the passive compression of the intravesical ureter’s ceiling on the detrusor muscle beneath it. This theory posits that the length and diameter of the intravesical part of the ureter are crucial for maintaining the closure of the VUJ and avoiding VUR [8].

Many researchers attribute VUR to the lateral positioning of the intravesical ostium and the relatively short transmural and submucosal part of the ureter compared to its diameter. Therefore, it is believed that the resolution of VUR spontaneously occurs as the bladder grows, lengthening the submucosal part [9].

Since the intravesical ureteric length-to-diameter ratio is lower than expected, it has been suggested that an intrinsic factor may impair the active antireflux mechanism. The active contraction of the longitudinal muscle layer in the ureter aids in moving urine into the bladder. Alterations in function and structure at the ureteric ends appear to compromise the active mechanism of the junction, leading to VUR [10,11].

Smooth muscle cells play a role in transforming the extracellular matrix (ECM) by producing proteinases and their inhibitors. Consequently, these cells significantly impact the maturation of refluxing ureters. The turnover of the ECM, particularly collagen I and III, involves matrix metalloproteinases (MMPs), which are produced by connective cells of mesenchymal origin, including fibroblasts, myoblasts, and CD68-positive macrophages [12].

The defect in the innervation of the distal ureteral ends is considered crucial for altering the active anti-reflux mechanism. Consistent and uniform peristaltic movement of the ureters is crucial in the process of transporting urine to the bladder. Many researchers have noted that dysplasia, atrophy, and disorganization of refluxing ureters are key factors in the failure of the active valve mechanism [13].

Grades of VUR 

The preferred method for assessing and grading VUR is direct cystography using a voiding cystourethrogram (VCUG). It is advisable to perform many VCUG imagings (at least two) to detect reflux, which can occur at different stages of the voiding cycle [14-16].

**Figure 1:**
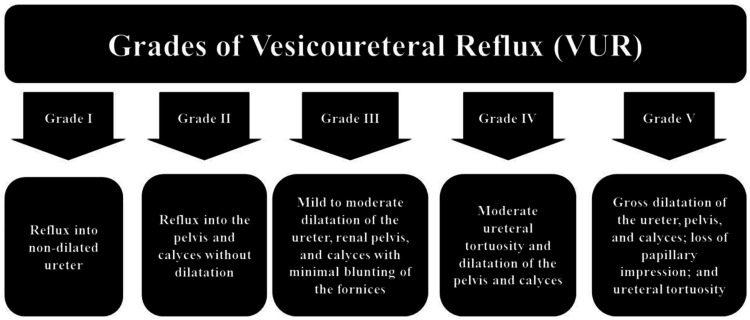
Grades of vesicoureteral reflux (VUR). This figure is original and created by the authors.

The VCUG results, combined with the patient's age and clinical presentation, are used to grade VUR and develop a treatment strategy. A global classification system for VUR was developed to support making clinical decisions. In grade 1, reflux is confined to the non-dilated ureter. In grade 2, reflux extends to the ureter and renal pelvis with no dilatation. Grade 3 involves reflux with mild dilatation of the ureters and pyelocalyceal system, accompanied by slight blunting of the calyces. In grade 4, the reflux involves a mild-to-moderate dilatation and tortuosity of the ureter, with calyceal blunting, yet the renal papilla remains visible. In grade 5, the reflux is marked by a severely dilated and highly tortuous ureter, large dilatation of the pelvicalyceal system, loss of renal fornices, and an absence of the papillary impression on imaging (Figure 1). Grades 3-5 are classified as dilating or high-grade VUR, which carry a higher risk of UTIs and other complications, particularly in grades 4 and 5 [17-19].

Pathophysiology of UTI in children with VUR

Pyelonephritis is typically caused by bacteria ascending through the urinary tract rather than spreading through the bloodstream. VUR and urine stasis make the renal pelvis more susceptible to infection. The parenchyma of the kidney can then be invaded, causing inflammation and edema. This inflammation compresses the small vessels of the parenchyma, potentially leading to ischemia, microabscesses, and necrosis. If the kidney parenchyma cannot heal from this damage, permanent kidney scarring with loss of the volume and function of the parenchyma may occur [20].

Refluxing sterile urine does not reduce kidney function, but the high-grade VUR is frequently linked to congenital renal dysplasia, which can impact kidney structure. The degree of kidney damage following an infection is influenced by factors related to the bacteria and host that determine the response to infection. Long-term consequences of kidney scarring, including hypertension, proteinuria, or chronic renal failure, may manifest later (in the second or third decades of life) [21].

Risk factors for recurrent UTI in children with VUR

According to a multisite prospective cohort study that was conducted to assess the risk factors for recurrent urinary tract infection and renal scarring, children with VUR have a high risk of febrile UTI recurrence over two years compared to those without VUR, with the highest rates observed in those with grade 3 or 4 VUR (Kaplan-Meier estimate: 28.9%) [22]. This cohort study also reported that the increased risk of recurrence became apparent within the first six months after enrolment in the trial and remained consistent [22]. In contrast, factors such as gender, age, type of initial UTI, and bladder and bowel dysfunction (BBD) at baseline were considered. The link between VUR and the recurrence of infection was stronger in children under 24 months and those with BBD at baseline. Univariable analyses identified additional factors linked to recurrent febrile UTIs, including age 36-71 months, white race, presence of BBD, a second UTI at enrollment, infection caused by Escherichia coli, parental education level, and kidney scarring on baseline dimercaptosuccinic acid (DMSA) scintigraphy. The multivariable model showed that only the grade and severity of VUR, presence of BBD from the beginning, and kidney scarring at baseline were significantly linked to recurrent UTI [22].

Evaluating and diagnosing VUR following febrile UTIs in children

The first evaluation after a febrile UTI aims to avoid the recurrence, prevent renal damage, and reduce morbidity. American Urological Association (AUA) and the European Association of Urology (EAU) guidelines for diagnosing and treating VUR suggest that, a thorough history, physical examination, and assessment of height, body mass index, blood pressure, and serum creatinine (to determine glomerular filtration rate) are necessary. Urine analysis for proteinuria and bacteriuria should be conducted, and if an infection is detected, a culture and sensitivity test should follow. Recent studies indicate that serum procalcitonin levels are linked to APN-related kidney injury, which appears on DMSA scans, and increased levels of ESR and CRP. Measuring procalcitonin levels helps identify clinically significant VUR and kidney injury, reducing the need for VCUG [23-25].

The guidelines from the EAU and the European Society of Pediatric Urology (ESPU) suggest using ultrasonography and VCUG for the first evaluation. In contrast, the American Academy of Pediatrics (AAP) suggests starting with USG alone and only resorting to VCUG if there are recurrent UTIs, ureteral dilation, or renal anomalies detected on the USG. The guidelines from the EAU, ESPU, and AUA recommend a technetium-99m Tc DMSA scan for grade 4 and 5 VUR, elevated creatinine levels, and concurrent UTIs [26-29]. The guidelines suggest a top-down approach, beginning with USG and DMSA scintigraphy to identify renal dysplasia and acquired renal scars. They endorse performing VCUG only if there is renal involvement, aiming to minimize urethral catheterization, reduce gonadal exposure to ionizing radiation, and avoid detecting clinically insignificant VUR. In addition to DMSA, 51Cr-ethylenediamine acetic acid has proven effective for assessing renal function. Magnetic resonance urography is also recommended for identifying renal hypotrophy [30].

Treatment of VUR and prevention of recurrent febrile UTI

Surgical Therapy of VUR

The primary aim of treating VUR is to shield the patient from UTIs, kidney damage, and related health issues. To achieve this, factors such as the patient’s age, gender, reflux severity, history of repeated UTIs, kidney function, and any related bladder-bowel dysfunction need to be assessed. Decisions should be made in consultation with family members. Options are conservative and interventional treatments. Surgical therapy is an option for poorly managed UTIs, high-grade VUR, kidney impairment detected initially or in the follow-up period, frequent UTI attacks in older populations not suited for CAP, and grades 4 and 5 VUR accompanied by lower urinary tract impairment [31]. Open surgery has been shown to be highly effective regardless of the method applied and is recommended for patients having urinary tract malformations and functional abnormalities, such as BBD and duplicate ureters. It is a well-established surgical treatment with proven long-term efficacy and a low rate of complications [32,33]. While circumcision does not directly treat VUR, it is considered an adjunctive strategy to reduce the risk of UTIs in male patients, especially those unresponsive to other preventive measures, such as prophylactic antibiotics. Studies have shown that circumcision can significantly reduce the incidence of recurrent UTIs in male patients with VUR. This is particularly relevant for patients with higher grades of VUR, where the risk of kidney involvement and subsequent renal scarring is elevated [32,33]. The laparoscopic transvesical Cohen’s technique boasts a success rate of 91-96%, which is on par with open surgery. Despite its effectiveness, the procedure is technically demanding and necessitates a steep learning curve. Potential complications include urinary leakage, anastomotic strictures, and hematuria. In addition, endoscopic injection therapy (e.g., Deflux) has a success rate of 70-90%, lower than surgical options but effective for lower grades of VUR. Robotic-assisted laparoscopic ureteral reimplantation enhanced precision, improved visualization, and reduced pain and scarring with a success rate of 94-99%, comparable to open surgery [34-36].

Continuous Antibiotic Prophylaxis (CAP)

CAP is commonly recognized as a groundbreaking non-invasive treatment for VUR as it helps prevent repeated febrile UTIs and subsequent new kidney scarring. Deciding between CAP and just observation depends on various factors, such as age, presence of febrile UTIs, dysfunction of the lower urinary tract, and constipation [7].

When selecting antimicrobials for CAP, it is crucial to consider the sensitivity of intestinal bacteria and the potential for antimicrobial resistance. In clinical practice, cephem antibiotics, penicillin antibiotics, and trimethoprim-sulfamethoxazole combinations are commonly used. Cephem antibiotics, particularly traditional ones, are frequently used and can be administered from early infancy in Japan. One oral dose, ranging from one-third to one-sixth of the standard pediatric daily dose, is recommended at bedtime [37]. Infants under two months old can receive penicillin as a single oral dose, equivalent to one-third of the recommended daily dose, administered at bedtime. TMP-SMX is regarded as a standard treatment option. However, it is contraindicated in infants under two months of age due to the potential for severe adverse effects. Careful monitoring through blood tests is essential [38].

Since BBD typically emerges following toilet training is completed, we present the uses for CAP before and after the establishment of voluntary micturition. BBD considerations are relevant only in the latter phase.

According to the period from birth, until toilet training is completed, VUR is sometimes detected through VCUG based on fetal and neonatal ultrasound findings in patients without febrile UTI. The 2010 consensus statement from the Society for Foetal Urology (SFU) recommends initiating CAP before confirming VUR and continuing it if it is confirmed. If febrile UTI is absent, CAP is advised for grade 3 or severe VUR, while it is elective for grade 1-2 VUR. However, for patients having febrile UTI, CAP is advised regardless of VUR severity, particularly in those under one year old. In patients aged one year or older, detecting BBD before toilet training is challenging. Therefore, these guidelines suggest CAP in grade 1-5 VUR patients who have not yet finished toilet training if febrile UTI is present [39,40].

It is rare for children after the age of toilet training to have no history of febrile UTIs or BBD. This is because both conditions are quite common in childhood. UTIs, especially febrile ones, are frequent in young children, and BBD often coexists with UTIs. CAP is advised in patients with grade 2-5 VUR who exhibit renal cortical abnormalities; in other cases, its use is discretionary. For patients with no febrile UTI but having BBD, it is essential to treat BBD. Given the high risk of febrile UTI in these patients, CAP is advised for patients with grade 3-5 VUR due to its anticipated effectiveness. For patients with febrile UTI but without BBD, CAP is considered a secondary option to surgical intervention for grade 3 or severe VUR. CAP is recommended only until surgery is performed or if surgery is not chosen. For grade 1-2 VUR, it is optional. For patients with both febrile UTI and BBD, CAP is advised for grade 1-5 VUR, with the primary focus on treating BBD. If surgery is needed, it will be the best option [39,40].

Efficacy of CAP in VUR 

Controversy exists over the use of CAP vs observation in the management of children with VUR. The reported effectiveness of CAP in children with reflux varies substantially. Pediatric trials on the prevention of UTIs have primarily involved those with nonexistent or low-grade VUR, mostly in girls who also had concomitant BBD [41-43]. Over 90% of the participants in the most recent prospective studies involved in the RIVUR experiment were female, and 80% had grade 2 or 3 VUR [7]. One of the included studies reported that antibiotic prophylaxis has not significantly affected the recurrence of UTI [44]. Nevertheless, other studies reported that prophylaxis considerably minimized the risk of UTI in certain children with VUR [45-47]. According to another study, boys who had grade 3 VUR (the highest grade in this study) had a significantly lower rate of recurrent UTIs [45]. In the RIVUR trial, recurrence rates were reduced by 80% and 60%, respectively, in children having baseline BBD and children having a febrile index infection regardless of several baseline characteristics, such as age, degree of VUR, or status of VUR throughout the study [48]. It also included renal scarring as a secondary objective and reported that antimicrobial prophylaxis did not significantly alter the outcome. Nevertheless, this does not cancel the benefit of continuous antibiotics in preventing kidney scars since they do not have adequate power to assess variations in renal scarring.

A meta-analysis involved young children following a first or second time of UTI. CAP was successful in preventing recurrent UTIs [49], but its use is beneficial only for high-grade VUR. Even so, it recommended the use of CAP irrespective of the degree of the reflux.

A recent multicenter, controlled trial evaluated and measured the effectiveness of CAP treatment given to infants with grade 3, 4, or 5 VUR prior to the development of any UTI. Compared to participants who were not treated, those who received CAP for 24 months had a 14.4% decreased incidence of a first symptomatic UTI, but it is not linked to a significant difference in the formation of new kidney scars at 24 months. Additionally, there was an elevation in the prevalence of non-E. coli organisms, such as pseudomonas and others, and an elevation in antibiotic resistance. There were no notable variations in the proportion of UTIs that required hospitalization between both groups [50].

The use of CAP is associated with decreasing the incidence of the first symptomatic UTI and preventing its recurrence but is not associated with decreasing the incidence of kidney scarring. Therefore, there are many questions about the intervention's therapeutic value, especially if its use is linked to dangerous adverse events, including antibiotic resistance.

Safety and possible long-term side effects of CAP

Antibiotics impact the gastrointestinal flora, which plays a vital role in enhancing digestion, energy turnover, metabolism, absorbing nutrients, pathogen defense, and the formation of the immune and nervous systems [51]. Antibiotic resistance has, regrettably, emerged as an unintended consequence of antibiotic use. As a result of antimicrobial resistance, the excessive use of antibiotics in pediatric urology, especially during CAP, and the lack of novel antimicrobial drugs have led to a worldwide problem. There is a 24-fold higher chance of E. coli resistance to TMP-SMX when CAP is used for UTIs [52]. Other trials have shown the growth of microorganisms other than E. coli exhibiting significant resistance rates [53]. Moreover, Costelloe et al. reported that extended antibiotic use is linked to an increased risk of developing antibiotic resistance [54]. They also observed that this treatment led to a pooled odds ratio of 2.5 for developing resistant UTI in two months from the initial treatment, which decreased to 1.3 in 12 months [54]. Other studies in pediatrics have observed links between antibiotic use and elevated body mass [55,56].

A review of electronic health records for 64,580 children in Philadelphia identified a higher risk of childhood obesity linked to broad-spectrum antimicrobial and repeated exposure [57].

There is growing evidence linking changes in gut microbiota to human metabolic processes [58]. Additionally, changes in gut flora have been linked to disruptions in the growing bone [59,60]. Researchers are increasingly identifying potential links between changes in the microbiome and the advancement of various diseases. Our knowledge of microbiome development, which starts in infancy, has expanded. Similar to how children achieve developmental milestones, the human microbiome also seems to follow its own set of milestones. Interruptions in this development, often due to antibiotic contact, can lead to enduring alterations in the structure and functionality of the gut microbiota [61,62].

Recent research indicates a potential link between taking antibiotics in the neonatal period and the development of childhood asthma, inflammatory bowel disease, and other immune-mediated disorders [63,64]. The comprehensive effects of antibiotics on metabolism, growth, and possible adverse events on immunity, the gastrointestinal tract, and the nervous system due to changes in the gut microbiota are still under investigation [55]. The following table presents the most used antibiotics for CAP and their side effects (Table 1).

**Table 1:** Safety of the most used antibiotics in patients with VUR. Abbreviations: TMP-SMX: Trimethoprim-sulfamethoxazole, CDAD: Clostridium difficile-associated diarrhoea. Data in this table are obtained from references [48,65-69].

Drug	Side effects	Long-term use outcomes	References
TMP-SMX	It is generally well-tolerated, but it can cause adverse effects, including gastrointestinal disturbances hypersensitivity reactions Hematologic abnormalities like neutropenia or thrombocytopenia.	Long-term use may increase the risk of antibiotic resistance.	[48]
Nitrofurantoin	Gastrointestinal side effects, such as nausea and vomiting, are more common.	Its long-term use can be associated with pulmonary toxicity, including chronic pulmonary fibrosis, although this is rare.	[65,66]
Cephalexin	It is generally safe with a low incidence of adverse effects. It can cause allergic reactions, including anaphylaxis in susceptible individuals. Gastrointestinal side effects, including diarrhoea, are relatively common.	CDAD can happen during or up to two months after starting cephalexin therapy. Oral thrush or a fresh yeast infection may arise from prolonged or frequent use.	[48,67]
Amoxicillin	It is well-tolerated, but common side effects include gastrointestinal upset, rash, and, in rare cases, CDAD.	Resistance to amoxicillin is a growing concern, especially with extended use.	[68]
Amoxicillin-Clavulanate	It can cause gastrointestinal issues, including diarrhoea and abdominal pain, due to the clavulanate component. As with other beta-lactams, allergic reactions can occur.	Skin reactions: Itching and rashes are possible Vaginal yeast infections can occur as a result of yeast infections.	[47,69]

Cost-effectiveness of CAP

There are different opinions that the risks of prophylaxis exceed the benefits [48]. A cost-utility analysis, which compares the cost per quality-adjusted life year (QALY) gained across different treatment regimens, is particularly well-suited to address this dispute. It was established to determine if prophylactic antibiotics use is of benefit for our population and, if not, which child subcategory would benefit most from prophylaxis [70]. They reported that, with an incremental cost-utility ratio (ICER) of $37,903 per QALY achieved, the use of only grade 4 VUR was determined to be the most economical option. Notably, these primary conclusions would still hold true if they had selected a different, widely used threshold (e.g., $50,000 or $200,000) [71-73]. Their findings also imply that the prolonged prophylactic antibiotics used in children having grades 1-3 VUR is unexpected to be deemed cost-effective [70].

Current guidelines regarding the use of CAP 

The 2023 update from the European guidelines on VUR in children highlighted that CAP is not required for all VUR children. The PREDICT trial found that CAP offers a modest but considerable advantage in protecting against the first attack of UTI in children with grade 3-5 VUR with no history of UTIs. However, this comes with an increased risk of non-E. coli and resistant infections. It is recommended to continue CAP til BBD is resolved. In children with febrile UTIs and grade 4-5 VUR, starting with medical management is recommended, with surgery considered only if there is noncompliance with CAP. Recurrent febrile UTIs, in spite of the use of CAP or the persistence of symptoms of VUR in the long-term follow-up period, are considered non-compliant patients with CAP [74].

The Indian Society of Pediatric Nephrology (ISPN) provides several recommendations for the utilization of prophylactic antibiotics for UTIs in children. They discourage using prophylactic antibiotics to prevent UTIs in children with normal tract anatomy and BBD. However, they suggest using prophylactic antibiotics to protect against repeated febrile UTIs in children with VUR of grades 3-5. It is not advised to use antibiotic prophylaxis to prevent symptomatic UTIs for children with antenatally detected hydronephrosis and are waiting for assessment. For children older than three months, cotrimoxazole or nitrofurantoin is recommended as the initial antibiotic used. Additionally, they recommend stopping prophylactic antibiotics in children over two years old when they are toilet trained, have no BBD, and have not had a febrile UTI in the past year [75].

Future directions and alternatives to CAP 

Intermittent Antibiotic Prophylaxis

The occurrence of UTIs when using continuous and intermittent prophylaxis did not vary noticeably in children having VUR. Therefore, the intermittent method may be used effectively with fewer adverse events in the management of these patients as a result of administering lower doses. This method is also considered cost-effective compared to using continuous prophylaxis [76].

Cranberry (Vaccinium Macrocarpon)

The cranberry subgenus is frequently used to protect against UTIs in women and has also been studied in the pediatric population. Studies have shown that children are more likely to comply with cranberry treatment than oral antibiotics, with fewer side effects. Even at low concentrations of 75 µg/mL, cranberry can be effective [47,77].

An RCT with 225 children observed no substantial decrease in UTI recurrence in those using cranberry juice. Notwithstanding, the results showed a drop in the number of recurrences and a reduced dependency on antibiotics [78].

Probiotics

Probiotics are live microorganisms that, when consumed in sufficient quantities, offer health benefits. They are traditionally employed to enhance or renew gastrointestinal microbiota, especially in cases of dysbiosis. The rationale for using it to prevent UTIs is based on the theory of microbial competition, which posits that disrupting the normal flora makes individuals more prone to severe infections. Probiotics work to reestablish a beneficial bacterial flora and create an environment resistant to infections. Most research on probiotics for UTI prevention has focused on adult women. There are currently limited studies on their use in children. One notable study assessed the topical application of probiotics in patients having neurogenic bladder. They found that instilling a benign strain of E. coli into the bladder reduced the incidence of recurrent UTIs [79,80].

Vaccination

Current vaccine research is focusing on specific virulence factors, including FimH or type 1 fimbriae. While no vaccines are present in the USA, some products, such as Uro-Vaxom, are available in Europe and Canada. Studies, primarily involving the adult population, have shown minimal prevention of developing UTIs. Though vaccine development is still in the early stages and no vaccines are available in the USA, this research is promising and could potentially offer a cutting-edge solution for patients with repeated UTIs in time to come [81].

## Conclusions

The use of CAP in managing VUR remains a contentious issue. CAP effectively reduces recurrent UTIs, particularly in children with high-grade VUR. However, its role in preventing long-term renal scarring is uncertain. Additionally, CAP carries potential drawbacks, including antibiotic resistance, adverse drug effects, and psychological stress on patients and families. Given these considerations, current evidence supports a more selective approach to CAP. Treatment decisions should be based on individual risk factors, such as the grade of VUR, prior UTI history, and the presence of renal abnormalities. For some patients, alternative strategies such as surgical intervention or non-antibiotic prophylaxis may provide better outcomes with fewer risks. CAP remains a valuable tool in certain cases but should not be used universally. Future research should focus on improving methods for identifying high-risk patients and developing new therapies to minimize dependence on long-term antibiotics.
